# Enhanced nigrostriatal neuron-specific, long-term expression by using neural-specific promoters in combination with targeted gene transfer by modified helper virus-free HSV-1 vector particles

**DOI:** 10.1186/1471-2202-9-37

**Published:** 2008-04-10

**Authors:** Haiyan Cao, Guo-rong Zhang, Xiaodan Wang, Lingxin Kong, Alfred I Geller

**Affiliations:** 1 Department of Neurology, West Roxbury VA Hospital/Harvard Medical School, W. Roxbury, MA 02132, USA

## Abstract

**Background:**

Direct gene transfer into neurons has potential for developing gene therapy treatments for specific neurological conditions, and for elucidating neuronal physiology. Due to the complex cellular composition of specific brain areas, neuronal type-specific recombinant gene expression is required for many potential applications of neuronal gene transfer. One approach is to target gene transfer to a specific type of neuron. We developed modified Herpes Simplex Virus (HSV-1) particles that contain chimeric glycoprotein C (gC) – glial cell line-derived neurotrophic factor (GDNF) or brain-derived neurotrophic factor (BDNF) proteins. HSV-1 vector particles containing either gC – GDNF or gC – BDNF target gene transfer to nigrostriatal neurons, which contain specific receptors for GDNF or BDNF. A second approach to achieve neuronal type-specific expression is to use a cell type-specific promoter, and we have used the tyrosine hydroxylase (TH) promoter to restrict expression to catecholaminergic neurons or a modified neurofilament heavy gene promoter to restrict expression to neurons, and both of these promoters support long-term expression from HSV-1 vectors. To both improve nigrostriatal-neuron specific expression, and to establish that targeted gene transfer can be followed by long-term expression, we performed targeted gene transfer with vectors that support long-term, neuronal-specific expression.

**Results:**

Helper virus-free HSV-1 vector packaging was performed using either gC – GDNF or gC – BDNF and vectors that contain either the TH promoter or the modified neurofilament heavy gene promoter. Vector stocks were injected into the midbrain proximal to the substantia nigra, and the rats were sacrificed at either 4 days or 1 month after gene transfer. Immunofluorescent costaining was performed to detect both recombinant gene products and nigrostriatal neurons. The combination of targeted gene transfer with neuronal-specific promoters improved nigrostriatal neuron-specific expression (83 to 93%) compared to either approach alone, and supported long-term (1 month) expression at levels similar to those observed using untargeted gene transfer.

**Conclusion:**

Targeted gene transfer can be used in combination with neuronal-specific promoters to achieve a high level of nigrostriatal neuron-specific expression. Targeted gene transfer can be followed by long-term expression. Nigrostriatal neuron-specific expression may be useful for specific gene therapy approaches to Parkinson's disease or for genetic analyses of nigrostriatal neuron physiology.

## Background

Gene transfer directly into neurons, using specific virus vectors, has potential for developing gene therapy treatments for specific neurological diseases and for studying neuronal physiology. However, due to the heterogeneous cellular composition of the brain, neuronal subtype-specific expression is required for many potential uses of neural gene transfer. The two predominate approaches are to target gene transfer to a specific cell type using a modified vector particle or to use a cell type-specific promoter to control expression [1-6]. A higher level of cell type-specific expression may be achieved by using these two complementary approaches together.

Helper virus-free HSV-1 vectors are attractive; they efficiently transduce neurons, have a large capacity, and cause minimal cytotoxicity [7-9]. The HSV-1 particle is composed of four components: i) The ~152 kb double stranded DNA genome is contained within ii) an icosahedral protein capsid, that is surrounded by iii) the tegument, a layer of proteins, and enclosed within iv) the envelope, a lipid bilayer containing 10 virally-encoded glycoproteins [10]. HSV-1 infection occurrs in two stages [11]. The initial binding to the cell surface is mediated by specific domains on glycoprotein C (gC) and gB that represent binding sites for glycosaminoglycans, principally heparin sulfate, present on cell surface proteoglycans [12-15]. Entry requires the subsequent binding of gD to a specific receptor. gD receptors include nectin-1 or nectin-2 of the immunoglobulin superfamily; the herpesvirus entry mediator (HVEM), which is a member of the tumor necrosis receptor family; and sites in heparin sulfate produced by specific isoforms of 3-*O*-sulfotransferases [11]. Entry occurs by fusion of the cell membrane and the viral envelope, and requires gB, gD, gH, and gL.

Targeted gene transfer with HSV-1 vectors was achieved by modifying gC to remove the heparin binding domain, and addition a binding site for a specific cell surface protein to either gC or gD. The initial study [16] reported a recombinant virus containing a chimeric gC – erythropoietin (EPO) that supported enhanced binding to cells that contained EPO receptors. Analogous designs used gD – IL13, or gD – urokinase plaminogen activator, or gD – single-chain anti-EGF receptor antibody chimeric proteins to target infection to tumor cells containing the cognate receptor [17-19]. Another study [20] used a HSV-1 plasmid (amplicon) vector expressing gC – His tag, and packaging with a helper virus deleted in gC; this strategy targeted infection to a cell line containing a pseudo-His-tag receptor. All of these reports [16-20] used specific HSV-1 viruses that grow productively and kill infected cells, confounding potential gene transfer studies.

We reported targeted gene transfer to a specific type of neuron in the brain [21]. Helper virus-free packaging was performed using pHSVlac [22] and either gC – glial cell line-derived neurotrophic factor (GDNF) or gC – brain-derived neurotrophic factor (BDNF) chimeric protein. Delivery of either vector stock into the midbrain supported 2.2 to 5.0-fold targeted gene transfer to nigrostriatal neurons [21], which contain both the GDNF receptor α-1 (GFRα-1 [23,24]) and the high-affinity BDNF receptor, TrkB[25,26]. Specifically, 75 to 80% of the transduced cells were nigrostriatal neurons, compared to 15–30% using untargeted gene transfer; and for the untargeted expression, 5 to 10% was in each of GABAergic neurons and glial fibrillary acidic protein-containing cells [21]. At times shortly after gene transfer, the HSV-1 immediate early (IE) 4/5 promoter in pHSVlac [22] supports expression in most cell types, which enabled an accurate assessment of the types of transduced cells. However, pHSVlac does not support significant levels of long-term expression in most neural cell types, and these rats were sacrificed at 4 days after gene transfer.

Specific promoters support long-term expression in neurons, or specific types of neurons, from HSV-1 vectors. To obtain long-term, neuronal-specific expression, we constructed a chimeric promoter containing a neurofilament heavy gene (NF-H) promoter, an upstream enhancer from the tyrosine hydroxylase (TH) promoter, and a β-globin insulator (INS-TH-NFH promoter [5]). Vectors containing the INS-TH-NFH promoter supported expression for 7, 8, or 14 months [27-29], and at 6 months, ~11,400 striatal neurons contained recombinant gene products (with 3 injection sites for gene transfer, [27]). Alternatively, vectors containing the TH promoter supported expression for at least 2 months in midbrain dopaminergic neurons, including nigrostriatal neurons (helper virus system [6,30]; helper virus-free system [31]). Of note, 40 to 60% of the transduced cells were nigrostriatal neurons [6,31], compared to 5% using pHSVlac [6]. Targeted gene transfer or use of the TH promoter each supported only partial nigrostriatal-specific expression, raising the possibility that further improvements in nigrostriatal-specific expression might be obtained by combining these complementary approaches.

Both to improve nigrostriatal neuron-specific expression, and to establish that targeted gene transfer can be followed by long-term expression, we performed targeted gene transfer using vectors that contain either the TH or INS-TH-NFH promoter. The combination of targeted gene transfer and a neuronal-specific promoter improved nigrostriatal neuron-specific expression to 83 to 93%. Of note, the levels of expression at 1 month were similar to those previously observed using untargeted gene transfer with each promoter.

## Results

### Vectors containing neuronal-specific promoters can be packaged using either gC – GDNF or gC – BDNF

The combination of targeted gene transfer with vectors that contain neuronal-specific promoters may support a higher level of nigrostriatal neuron-specific expression than either approach alone. Because gC – GDNF or gC – BDNF supported a similar level of targeted gene transfer to nigrostriatal neurons (75 to 80%) at 4 days after gene transfer [21], we decided to examine each construct. To explore the potential for targeted gene transfer to be followed by long-term expression, we decided to test two promoters that can support long-term expression in nigrostriatal neurons, the TH or INS-TH-NFH promoters [5,6]. We arbitrarily chose the combinations of gC – BDNF targeting with a vector containing the TH promoter (pTHlac/gC – bdnf), or gC – GDNF targeting with a vector containing the INS-TH-NFH promoter (pINS-TH-NFHlac/gC – gdnf).

We quantified the additional nigrostriatal neuron-specific expression supported by the combination of targeted gene transfer using a neuronal-specific promoter by comparison to a pHSVlac-type vector. At times shortly after gene transfer, pHSVlac-type vectors, which contain the HSV-1 IE 4/5 promoter, support expression in most types of neural cells. To enable comparisons within the same rats to vectors that express β-galactosidase (β-gal), we used pHSVpkcΔGG. pHSVpkcΔGG expresses a flag-tagged catalytic domain of protein kinase C (PKC), and contains a point mutation that abolishes protein kinase activity [32].

**Figure 1:**
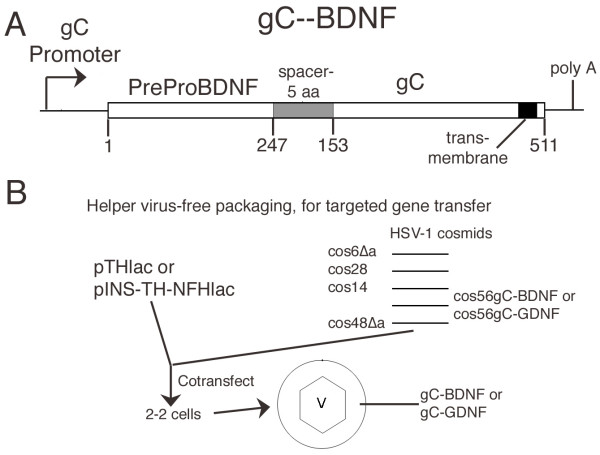
Schematic diagrams of (A) the gC – BDNF construct, or (B) helper virus-free packaging for targeted gene transfer. **A**. gC – BDNF contains the gC promoter, a BDNF cDNA (preproBDNF, aa 1–247), a spacer of 5 aa, and a deletion of gC containing aa 153 to the C-terminus (aa 511) [21]. Expression of gC – BDNF will be regulated similar to the wt gC gene, as this construct retained the gC promoter and polyadenylation site. BDNF, a secreted protein, contains a signal sequence [46] to support proper posttranslational processing, and the gC deletion retains the transmembrane domain to support insertion into the envelope of HSV-1 particles. **B**. Helper virus-free packaging [7, 50] was performed using either pTHlac or pINS-TH-NFHlac and either gC – BDNF or gC – GDNF. A HSV-1 vector and a HSV-1 cosmid set that contained a modified gC construct and lacks an **a **sequence (contains the packaging site) were cotransfected into 2-2 cells [51]. The resulting HSV-1 vector particles should contain the vector, modified gC, and all the other HSV-1 envelope glycoproteins. Thus, these vector particles are designed to enable initial binding to either BDNF or GDNF receptors on the surface of cells, followed by entry using the same mechanism as used by wt HSV-1 particles [11].

These vectors were packaged into HSV-1 particles for targeted gene transfer (Figure 1). The titers obtained for pTHlac/gC – bdnf or pINS-TH-NFHlac/gC – gdnf were similar to the titers obtained for pHSVpkcΔGG/gC – bdnf or pHSVpkcΔGG/gC – gdnf (titers in methods), or the titers previously reported for either pHSVlac/gC – bdnf or pHSVlac/gC – gdnf [21]. We previously showed that gC – GDNF or gC – BDNF are each incorporated into HSV-1 virus particles [21], and we did not perform analogous Western blot assays in this study. For clarity of presentation, we first present results on the cell type specificity of expression supported by pTHlac/gC – bdnf, followed by the cell type specificity observed using pINS-TH-NFHlac/gC – gdnf, and then the data on long-term expression.

### In the rat brain, pTHlac/gC – bdnf supported higher levels of nigrostriatal neuron-specific expression than pHSVpkcΔGG/gC – bdnf

Due to the small extent of the substantia nigra pars compacta (SNc) in the dorsal-ventral dimension, and the heterogeneous cellular composition of the midbrain, injections of HSV-1 vectors into the midbrain near the substantia nigra typically support gene transfer to different populations of cells in different rats, complicating comparisons between rats. To control for the variability in injection sites between rats, and to quantify the additional nigrostriatal neuron-specific expression supported by the TH promoter after targeted gene transfer, we co-injected mixtures of equal titers of pTHlac/gC – bdnf and pHSVpkcΔGG/gC – bdnf (see methods for titers and injection conditions). The rats were sacrificed at either 4 days or 1 month after gene transfer, the brains were sectioned, and alternating sections were costained for either β-gal-immunoreactivity (IR) and TH-IR, or flag-IR (PkcΔGG contains the flag tag) and TH-IR. Nigrostriatal neurons contain TH [33], enabling identification using an anti-TH antibody.

**Figure 2:**
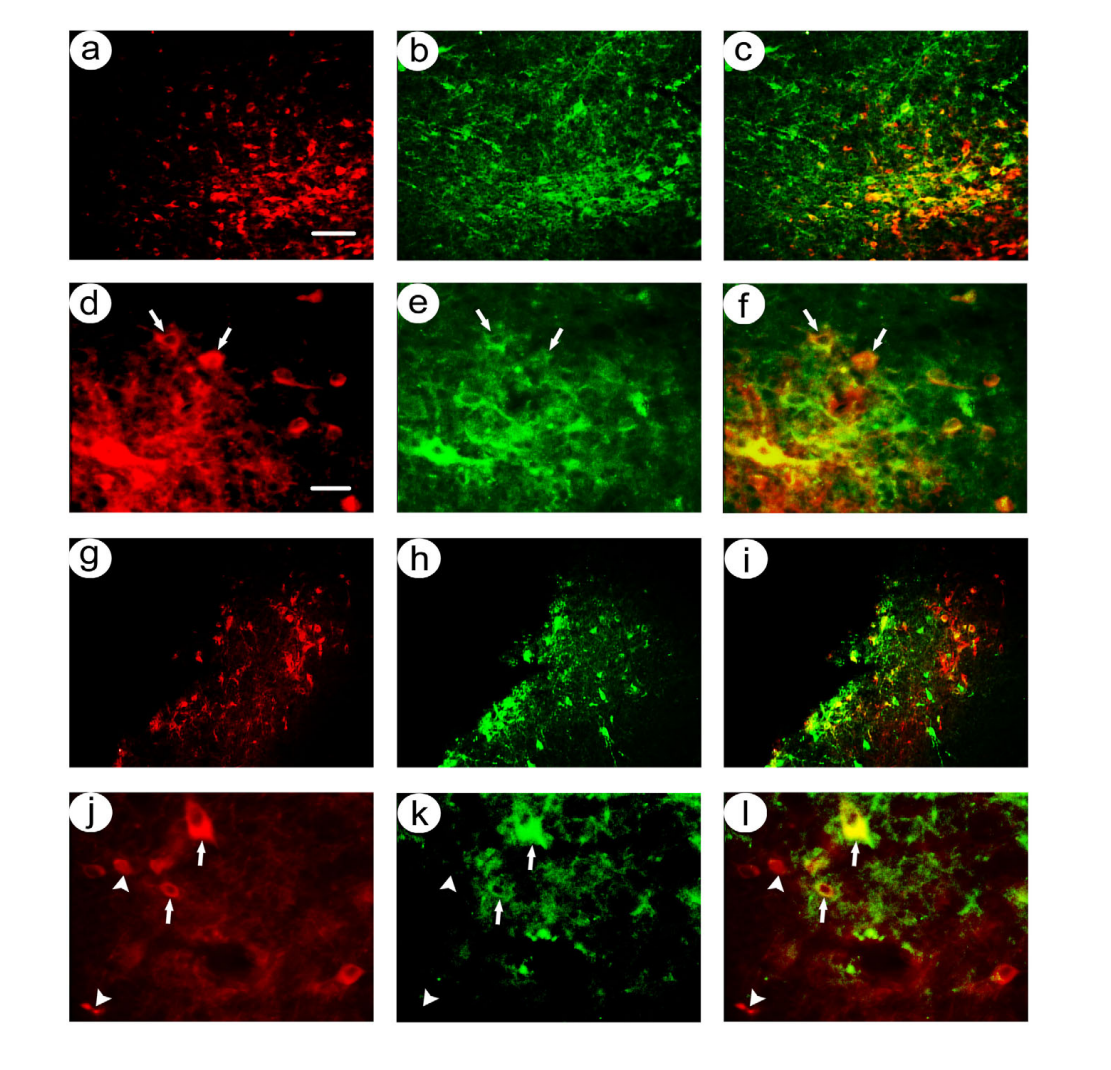
Costaining for nigrostriatal neuron-specific recombinant gene expression in rats sacrificed at 4 days after co-injection of pTHlac/gC – bdnf and pHSVpkcΔGG/gC – bdnf into the midbrain. Alternating sections were costained using either rabbit anti-β-gal and mouse anti-TH or mouse anti-flag (detects PkcΔGG) and rabbit anti-TH, and these antibodies were visualized using rhodamine- or fluorescein-conjugated secondary antibodies. **A-F**. pTHlac/gC – bdnf supported expression of β-gal in nigrostriatal neurons: Low power (A-C); β-gal-IR (A), TH-IR (B), and merge (C). High power (D-F); β-gal-IR (D), TH-IR (E), and merge (F). Arrows indicate examples of costained cells. **G-I**. pHSVpkcΔGG/gC – bdnf supported expression of β-gal in nigrostriatal neurons, with some expression in cells that lacked TH-IR: Low power (G-I); flag-IR (G), TH-IR (H), and merge (I). High power (J-L); β-gal-IR (D), TH-IR (E), and merge (F). Arrowheads indicate examples of flag-IR only. Scale bars: (A-C and G-I) 100 μm, (D-F and J-L) 30 μm.

At 4 days after gene transfer, low power photomicrographs showed that most of the β-gal-IR cells were located in a narrow band that contained TH-IR neurons in the SNc (Figure 2A–C; pTHlac/gC – bdnf expresses β-gal), and a few β-gal-IR cells were located ventral to the TH-IR cells. High power views showed that the clear majority of the β-gal-IR cells also contained TH-IR (Figure 2D–F). In contrast, while many of the flag-IR cells were located within the band of TH-IR cells, more of the flag-IR cells were located either dorsal or ventral to the TH-IR cells (Figure 2G–I; pHSVpkcΔGG/gC – bdnf expresses PkcΔGG, which contains the flag-tag). High power views showed some that flag-IR cells contained TH-IR, and some flag-IR cells lacked TH-IR (Figure 2J–L). Cell counts from 4 rats (Table 1) showed that an average of 84 ± 1% (mean ± sem) of the β-gal-IR cells also contained TH-IR, but only 70 ± 2% of the flag-IR cells also contained TH-IR.

**Table 1:** The numbers of β-gal or PkcΔGG positive cells that contained TH-IR, in rats sacrificed at 4 days after co-injection of pTHlac/gC – bdnf and pHSVpkcΔGG/gC – bdnf into the midbrain

	**Numbers of positive cells**		**%**	**Numbers of positive cells**		**%**
**Rat**	**β-gal-IR**	**TH-IR**	**Costained**	**Flag-IR**	**TH-IR**	**Costained**
1	347	287	83	328	232	71
2	322	278	86	205	134	66
3	340	274	81	470	354	75
4	418	358	86	310	212	68
Average			84 ± 1			70 ± 2

A mixture of equal IVP/ml of pTHlac/gC – bdnf and pHSVpkcΔGG/gC – bdnf (for titers and injection conditions, see methods) was injected into the midbrain proximal to the substantia nigra. The rats were perfused at 4 days after gene transfer, the brains were sectioned, and alternating sections were costained for either β-gal-IR or flag-IR and TH-IR. Sufficient sections were examined to count at least 200 cells that contained either β-gal-IR or flag-IR in each rat. In the sections that were examined, all of the β-gal-IR or flag-IR cells were scored for containing or lacking TH-IR. For the average % costaining, the means ± sems are shown.

**Figure 3:**
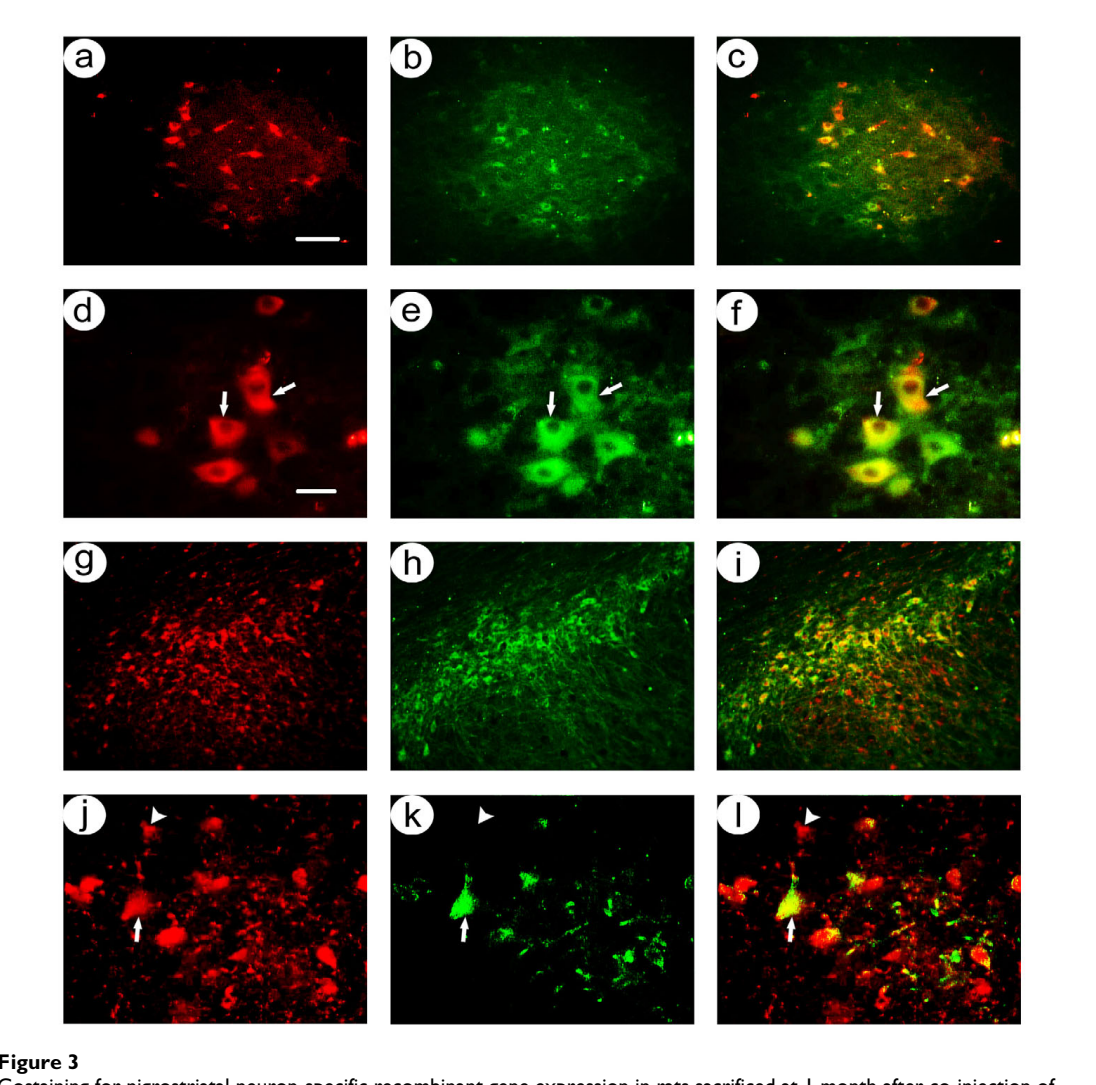
Costaining for nigrostriatal neuron-specific recombinant gene expression in rats sacrificed at 1 month after co-injection of pTHlac/gC – bdnf and pHSVpkcΔGG/gC – bdnf into the midbrain. Alternating sections were costained using either anti-β-gal and anti-TH, or anti-flag (detects PkcΔGG) and anti-TH. **A-F**. pTHlac/gC – bdnf supported expression of β-gal in nigrostriatal neurons: Low power (A-C); β-gal-IR (A), TH-IR (B), and merge (C). High power (D-F); β-gal-IR (D), TH-IR (E), and merge (F). Arrows indicate examples of costained cells. **G-I**. pHSVpkcΔGG/gC – bdnf supported expression of β-gal in nigrostriatal neurons, with some expression in cells that lacked TH-IR: Low power (G-I); flag-IR (G), TH-IR (H), and merge (I). High power (J-L); β-gal-IR (D), TH-IR (E), and merge (F). Arrowheads indicate examples of flag-IR only. Scale bars: (A-C and G-I) 100 μm, (D-F and J-L) 30 μm.

At 1 month after gene transfer, low power photomicrographs showed that almost all of the β-gal-IR cells were located within the area of TH-IR neurons in the SNc (Figure 3A–C), and high power views showed that these β-gal-IR cells also contained TH-IR (Figure 3D–F). In contrast, although numerous flag-IR cells were located within the band of TH-IR cells, many flag-IR cells were located either dorsal or ventral to the TH-IR cells (Figure 3G–I), similar to the results from rats sacrificed at 4 days. High power views showed that some flag-IR cells contained TH-IR, and some flag-IR cells lacked TH-IR (Figure 3J–L). Cell counts from 2 rats (Table 2; there were technical issues with perfusing 1 rat) showed that an average of 93 ± 1% of the β-gal-IR cells also contained TH-IR, but only 72 ± 3% of the flag-IR cells also contained TH-IR. Statistical analysis showed that significantly more β-gal-IR than flag-IR cells contained TH-IR (condition p < 0.0001, sacrifice time p < 0.05, 2-way ANOVA).

**Table 2:** The numbers of β-gal or PkcΔGG positive cells that contained TH-IR, in rats sacrificed at 1 month after co-injection of pTHlac/gC – bdnf and pHSVpkcΔGG/gC – bdnf into the midbrain

	**Numbers of positive cells**		**%**	**Numbers of positive cells**		**%**
**Rat**	**β-gal-IR**	**TH-IR**	**Costained**	**Flag-IR**	**TH-IR**	**Costained**
1	201	184	92	129	89	69
2	183	171	93	110	82	75
Average			93 ± 1			72 ± 3

This experiment was performed as in Table 1, except the rats were perfused at 1 month after gene transfer.

We previously showed that pretreatment of a pHSVlac/gC – bdnf stock with an anti-BDNF antibody reduced targeted gene transfer to nigrostriatal neurons to ~16%, a level similar to that obtained using untargeted gene transfer [21]. Analogous studies were not repeated using the current vector stocks.

### pINS-TH-NFHlac/gC – gdnf supported higher levels of nigrostriatal neuron-specific expression than pHSVpkcΔGG/gC – gdnf

**Figure 4:**
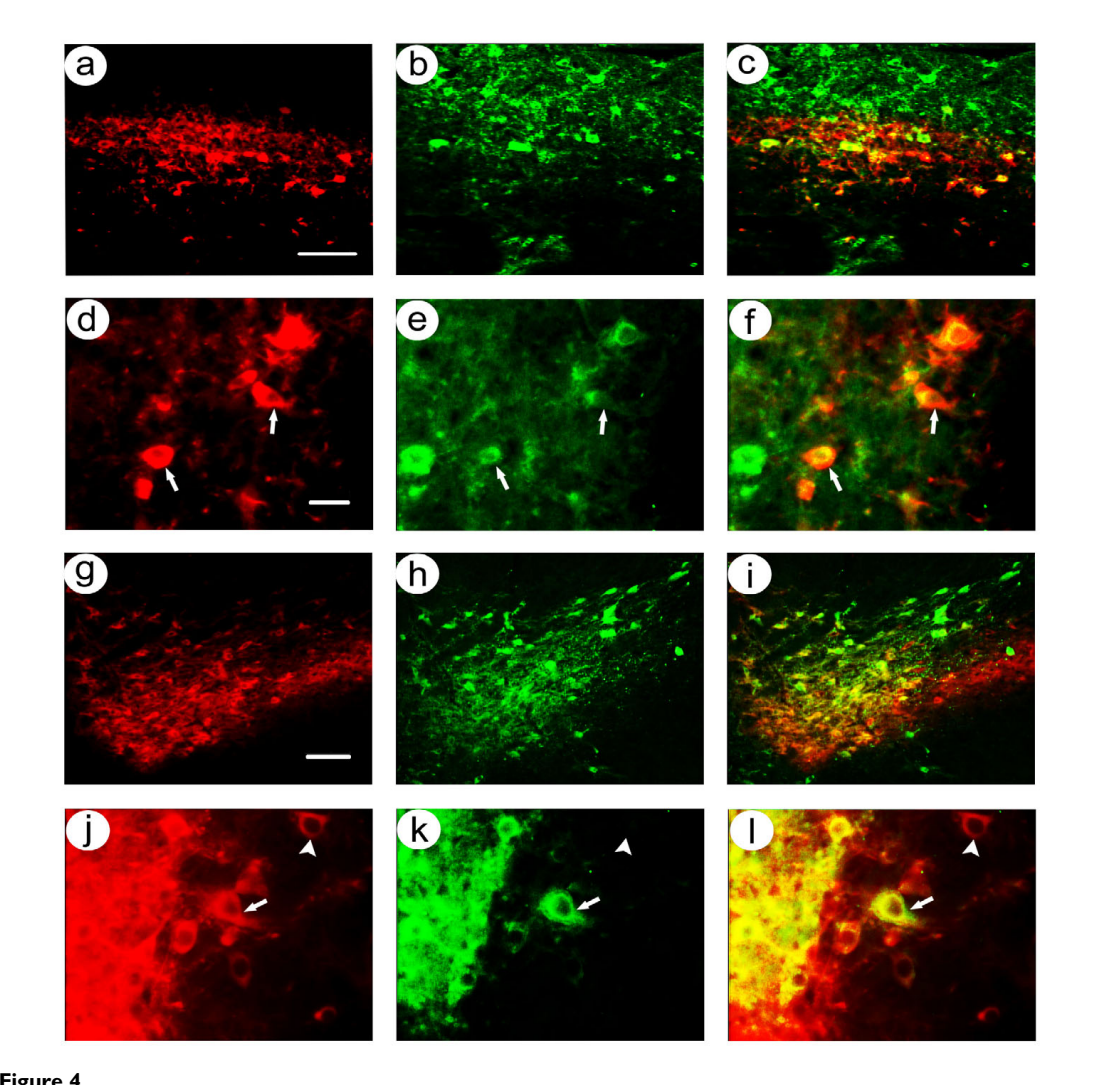
Costaining for nigrostriatal neuron-specific recombinant gene expression in rats sacrificed at 4 days after co-injection of pINS-TH-NFHlac/gC – gdnf and pHSVpkcΔGG/gC – gdnf into the midbrain. Alternating sections were costained using either anti-β-gal and anti-TH, or anti-flag and anti-TH. **A-F**. pINS-TH-NFHlac/gC – gdnf supported expression of β-gal in nigrostriatal neurons: Low power (A-C); β-gal-IR (A), TH-IR (B), and merge (C). High power (D-F); β-gal-IR (D), TH-IR (E), and merge (F). Arrows indicate examples of costained cells. **G-I**. pHSVpkcΔGG/gC – gdnf supported expression of β-gal in nigrostriatal neurons, with some expression in cells that lacked TH-IR: Low power (G-I); flag-IR (G), TH-IR (H), and merge (I). High power (J-L); β-gal-IR (D), TH-IR (E), and merge (F). Arrowheads indicate examples of flag-IR only. Scale bars: (A-C) 100 μm, (G-I) 100 μm, (D-F and J-L) 30 μm.

We co-injected mixtures of equal titers of pINS-TH-NFHlac/gC – gdnf and pHSVpkcΔGG/gC – gdnf into the midbrain proximal to the SNc, and the rats were sacrificed at either 4 days or 1 month after gene transfer. At 4 days after gene transfer, low power photomicrographs showed that most of the β-gal-IR cells were located within the area of TH-IR neurons (Figure 4A–C), and low numbers of β-gal-IR cells were located ventral to the TH-IR cells. High power views showed that most of these β-gal-IR cells also contained TH-IR (Figure 4D–F). In contrast, many of the flag-IR cells were located within the band of TH-IR cells, and many of the flag-IR cells were located ventral to the TH-IR cells (Figure 4G–I). Consistent with the low power views, high power views showed that some flag-IR cells contained TH-IR, and other flag-IR cells lacked TH-IR (Figure 4J–L). Cell counts from 3 rats (Table 3) showed that an average of 83 ± 2% of the β-gal-IR cells also contained TH-IR, but only 68 ± 2% of the flag-IR cells also contained TH-IR.

**Table 3:** The numbers of β-gal or PkcΔGG positive cells that contained TH-IR, in rats sacrificed at 4 days after co-injection of pINS-TH-NFHlac/gC – gdnf and pHSVpkcΔGG/gC – gdnf into the midbrain

	**Numbers of positive cells**		**%**	**Numbers of positive cells**		**%**
**Rat**	**β-gal-IR**	**TH-IR**	**Costained**	**Flag-IR**	**TH-IR**	**Costained**
1	471	378	80	535	344	64
2	758	625	83	652	467	72
3	444	384	87	474	316	67
Average			83 ± 2			68 ± 2

This experiment was performed as in Table 1, except a mixture of equal titers of pINS-TH-NFHlac/gC – gdnf and pHSVpkcΔGG/gC – gdnf was injected into the midbrain.

**Figure 5:**
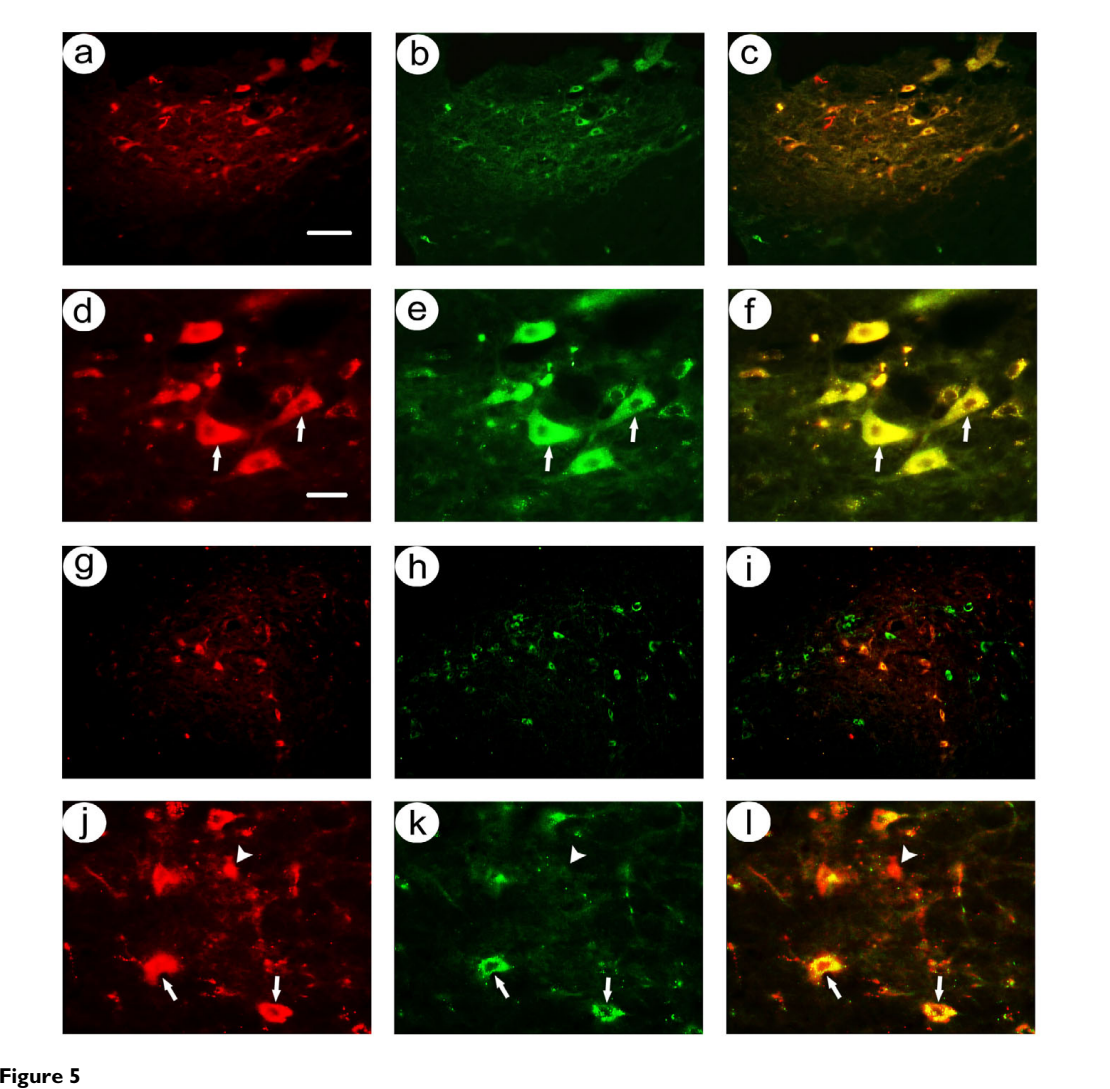
Costaining for nigrostriatal neuron-specific recombinant gene expression in rats sacrificed at 1 month after co-injection of pINS-TH-NFHlac/gC – gdnf and pHSVpkcΔGG/gC – gdnf into the midbrain. Alternating sections were costained using either anti-β-gal and anti-TH, or anti-flag and anti-TH. **A-F**. pINS-TH-NFHlac/gC – gdnf supported expression of β-gal in nigrostriatal neurons: Low power (A-C); β-gal-IR (A), TH-IR (B), and merge (C). High power (D-F); β-gal-IR (D), TH-IR (E), and merge (F). Arrows indicate examples of costained cells. **G-I**. pHSVpkcΔGG/gC – gdnf supported expression of β-gal in nigrostriatal neurons, with some expression in cells that lacked TH-IR: Low power (G-I); flag-IR (G), TH-IR (H), and merge (I). High power (J-L); β-gal-IR (D), TH-IR (E), and merge (F). Scale bars: (A-C and G-I) 100 μm, (D-F and J-L) 30 μm

At 1 month after gene transfer, low power photomicrographs showed that the area of β-gal-IR cells closely followed the area of TH-IR neurons in the SNc (Figure 5A–C), and high power views showed that these β-gal-IR cells also contained TH-IR (Figure 5D–F). In contrast, many flag-IR cells were located within the band of TH-IR cells, and some flag-IR cells were located either dorsal or ventral to the TH-IR cells (Figure 5G–I). High power views showed flag-IR cells that either contained or lacked TH-IR (Figure 5J–L). Cell counts from 3 rats (Table 4) showed that an average of 92 ± 0% of the β-gal-IR cells also contained TH-IR, but only 69 ± 5% of the flag-IR cells also contained TH-IR. Statistical analysis showed that significantly more β-gal-IR than flag-IR cells contained TH-IR (condition p < 0.001, sacrifice time p > 0.05, 2-way ANOVA).

**Table 4:** The numbers of β-gal or PkcΔGG positive cells that contained TH-IR, in rats sacrificed at 1 month after co-injection of pINS-TH-NFHlac/gC – gdnf and pHSVpkcΔGG/gC – gdnf into the midbrain

	**Numbers of positive cells**		**%**	**Numbers of positive cells**		**%**
**Rat**	**β-gal-IR**	**TH-IR**	**Costained**	**Flag-IR**	**TH-IR**	**Costained**
1	240	223	93	156	122	78
2	144	132	92	148	88	60
3	228	210	92	82	56	68
Average			92 ± 0			69 ± 5

This experiment was performed as in Table 1, except a mixture of equal titers of pINS-TH-NFHlac/gC – gdnf and pHSVpkcΔGG/gC – gdnf was injected into the midbrain, and the rats were perfused at 1 month after gene transfer.

### Following targeted gene transfer, pTHlac or pINS-TH-NFHlac supported long-term expression

The cell counts in Tables 1, 2, 3, 4 were used to calculate the total numbers of β-gal-IR cells supported by each vector, at either 4 days or 1 month after gene transfer. The results (Table 5) showed that the INS-TH-NFH or TH promoter each supported 37% of the number of cells at 1 month compared to 4 days. This 37% stability of long-term expression is similar the stabilities of expression observed following standard, untargeted gene transfer using either pTHlac [6,31] or pINS-TH-NFHlac [5]. Surprisingly, the IE 4/5 promoter supported 23 to 28% stability of expression in nigrostriatal neurons. In contrast, using untargeted gene transfer, pHSVlac supports ≤ 1% stability of expression at 1 month in the striatum, hippocampus, or specific neocortical areas [7,34,35]. Congruently, transgenic mice that contain specific HSV-1 IE promoters controlling a reporter gene showed that specific IE promoters support expression in only a few specific types of neurons [36-38]. Thus, the IE 4/5 promoter in pHSVlac-type vectors appears to selectively support higher levels of long-term expression in nigrostriatal neurons than in most other types of neurons, including those in the midbrain, striatum, or hippocampus [7,34,35].

**Table 5:** pTHlac or pINS-TH-NFHlac support expression for 1 month after targeted gene transfer to nigrostriatal neurons using either gC – bdnf or gC – gdnf, respectively

		**Numbers of positive cells**				% Long-term expression*	
		**4 days**		**1 month**			
**Vectors**	**gC**	**β-gal-IR**	**Flag-IR**	**β-gal-IR**	**Flag-IR**	**β-gal**	**Flag**
pTHlac & pHSVpkcΔGG	gC – bdnf	2,071 ± 602	1,723 ± 284	768 ± 36	478 ± 38	37	28
pINS-TH-NFHlac & pHSVpkcΔGG	gC – gdnf	2,230 ± 402	2,214 ± 209	816 ± 121	515 ± 94	37	23

Using pTHlac & pHSVpkcΔGG, 4 rats were analyzed at 4 days, and 2 rats were analyzed at 1 month. Using pINS-TH-NFHlac & pHSVpkcΔGG, 3 rats were analyzed each time point. The means ± sems are shown.

*% long-term expression = β-gal-IR cells at 1 month/β-gal-IR cells at 4 days × 100.

## Discussion

The combination of targeted gene transfer with a vector that contains a neuronal-specific promoter improved nigrostriatal neuron-specific expression, compared to either approach alone. Untargeted gene transfer with pHSVlac-type vectors supported 5 to 30% nigrostriatal neuron-specific expression [6,21]; pHSVlac-type vectors contain the HSV-1 IE 4/5 promoter that is active in multiple types of neural cells at times shortly after gene transfer. pTHlac, with standard untargeted gene transfer, supported 40 to 60% nigrostriatal neuron-specific expression [6,31]. Targeted gene transfer with pHSVlac-type vectors supported 68 to 80% nigrostriatal neuron-specific expression (this study and [21]). Targeted gene transfer with vectors containing the TH or INS-TH-NFH promoter supported 83 or 84% nigrostriatal neuron-specific expression at 4 days after gene transfer, and 92 or 93% nigrostriatal neuron-specific expression at 1 month after gene transfer. The ~10% increase in nigrostriatal neuron-specific expression between 4 days and 1 month after gene transfer may be due to a higher level of inappropriate expression from the neuronal-specific promoter at 4 days, before chromatin structure on the vectors has reached steady levels.

Targeted gene transfer with each of the neuronal-specific promoters supported similar levels of nigrostriatal neuron-specific expression, although the TH promoter is specific for nigrostriatal neurons while the INS-TH-NFH promoter is active in most types of neurons. Using targeted gene transfer with pHSVlac-type vectors, we showed that ~5% of the gene transfer was to glutamic acid decarboxylase (GAD)-IR neurons and [21], and GABAergic neurons in the substantia nigra pars reticulata are adjacent to the SNc. Also, using targeted gene transfer with pHSVlac-type vectors, we showed that 5 to 12% of the gene transfer was to glial fibrillary acidic protein (GFAP)-IR cells [21], and we did not examine gene transfer to other types of glia. Thus, inappropriate gene transfer to glla appears to account for more of the inappropriate gene transfer than gene transfer to other types of neurons, and both the TH and INS-TH-NFH promoters are neuronal-specific promoters that will reduce the inappropriate expression in glia.

Targeted gene transfer with either gC – GNDF or gC – BDNF was not complete, and some gene transfer to inappropriate cell types was observed (this study and [21]). The fragment of gC in either gC – GDNF or gC – BDNF lacks the heparin sulfate binding domain [14,39]; but the vector particles still contain gB, which contains a heparin sulfate binding domain [15,40]. In the context of HSV-1 viruses, deletion of either the gC or gB heparin sulfate binding domain reduced binding to fibroblast cells ~65% or ~20%, respectively, and deletion of both domains was approximately additive, reducing binding ~80% [15]. Thus, use of both a gB lacking a heparin sulfate binding domain (gBpK^- ^[15]) and a chimeric gC might improve targeted gene transfer to nigrostriatal neurons.

Using pHSVlac-type vectors, gC – GNDF or gC – BDNF appear to support similar levels of targeted gene transfer (this study and [21]); although over 20 rats have been analyzed, these are comparisons between different rats that do not control for variability in injection sites between rats. The untargeted gene transfer supported by the wt gB in these vector particles may be a larger variable than any differences in targeting efficiency between gC – GNDF or gC – BDNF. Of note, GDNF is more effective than BDNF in protecting nigrostriatal neurons in the 6-OHDA rat model of Parkinson's disease [29]. However, neuroprotection requires intracellular signaling, whereas targeted gene transfer requires only binding of the vector particle to the appropriate receptor on the cell surface. As nigrostriatal neurons contain high levels of both the GDNF receptor α-1 (GFRα-1 [23,24]) and the high-affinity BDNF receptor, TrkB [25,26], gC – GNDF or gC – BDNF may support similar efficiencies of targeted gene transfer to nigrostriatal neurons.

Following targeted gene transfer, the TH or INS-TH-NFH promoters supported levels of long-term expression that were similar to those observed following untargeted, standard gene transfer. gC – GNDF or gC – BDNF likely alter only the initial binding to cells, After the initial binding to cells, entry occurs by fusion of the cell membrane and the viral envelope, and requires gB, gD, gH, and gL. Thus, using either targeted gene transfer or standard gene transfer, entry, vector particle uncoating, and gene expression are likely to occur by similar pathways.

## Conclusion

Our results establish that following targeted gene transfer, specific promoters can support long-term expression from HSV-1 vectors. Targeted gene transfer to nigrostriatal neurons, followed by long-term expression, may be useful for specific gene therapy treatments or physiological studies. In relation to Parkinson's disease, clinical trials with recombinant GDNF protein were halted due to side effects [41-43], suggesting that more restricted expression of GDNF is desirable. In relation to studies on neuronal physiology, transgenic mice that use the TH promoter to control recombinant gene expression will alter expression in most catecholaminergic neurons, whereas the approach described here can support a selective analysis of nigrostriatal neuron physiology. More generally, targeted gene transfer followed by long-term expression may have specific clinical or basic neuroscience applications in brain areas that contain a complex mixture of different types of neurons, including the midbrain, and possibly also specific forebrain areas.

## Methods

### Materials

Dulbecco's modified minimal essential medium, fetal bovine serum, G418, lipofectamine, and OPTI-MEM I were obtained from Invitrogen. 5-bromo-4-chloro-3-indoyl-β-D-galactopyranoside (X-Gal) was obtained from Sigma. Mouse anti-E. coli β-gal and mouse monoclonal anti-flag were obtained from Sigma, and rabbit anti-TH was obtained from Chemicon. Rhodamine isothiocyanate-conjugated goat anti-mouse immunoglobulin (Ig) G and fluorescein isothiocyanate-conjugated goat anti-rabbit IgG were obtained from Jackson ImmunoResearch Laboratories.

### Cosmids and vectors

The HSV-1 genome is represented by cosmid set C (cos6, cos14, cos28, cos48, cos56, [44]); the **a **sequence was deleted from the two cosmids that contained it (cos6Δ**a**, cos48Δ**a **[7]). cos56 gC – GNDF and cos56 gC – BNDF have been described [21]. These chimeric gC (Figure 1) deleted aa 1–152 of gC, removing the heparin sulfate binding domain [12-14], required for the initial attachment of HSV-1 particles to the cell surface. This deletion retained the transmembrane and internal domains to support insertion of the chimeric proteins into the HSV-1 particle envelope. A GDNF cDNA or a BDNF cDNA was inserted inframe at the N-terminus of the gC deletion, with 5 aa spacer separating the two domains; preproGDNF is 211 aa and mature GDNF is 134 aa [45], or preproBDNF is 247 aa and mature BDNF is 119 aa [46]. GDNF or BDNF each contain a signal sequence [45,46], to support posttranslational processing of the chimeric protein. These constructs retained the gC promoter and polyadenylation site, enabling expression to be regulated similar to wt gC.

HSV-1 vectors that express the Lac Z gene from either a 6.8 kb fragment of the TH promoter (pTHlac [6]) or a modified neurofilament promoter (pINS-TH-NFHlac [5]) have been described. The INS-TH-NFH promoter contains a mouse NF-H promoter (0.6 kb fragment from plasmid pH-615 [47]), upstream sequences from the rat TH promoter (-0.5 kb to -6.8 kb [48]), and the chicken β-globin INS (1.2 kb [49]). pHSVpkcΔGG [32] contains the pkcΔGG gene under the control of the HSV-1 IE 4/5 promoter. PkcΔ is a flag-tagged, catalytic domain of rat PKC βII, and PkcΔGG contains a point mutation in an evolutionarily conserved lys residue that abolishes PKC enzyme activity [32].

### Cells, vector packaging, and titering

The growth of baby hamster kidney fibroblast (BHK21) cells [50] and 2-2 cells [51] have been described. Helper virus-free packaging [7] was performed using a modified protocol [50] that improves the efficiency. 2-2 cells were cotransfected with a vector and a HSV-1 cosmid set that lacked a packaging site (cos6Δ**a**, cos14, cos28, cos48Δ**a**, and one of two cos56 (containing gC – GNDF or gC – BNDF)). Vector stocks were purified [52].

Vector stocks were titered by counting the numbers of positive cells at 1 day after transduction of BHK fibroblast cells. Vectors that express β-gal were titered by staining with X-gal, and pHSVpkcΔGG stocks were titered using an anti-flag antibody [32]. Vector stocks were titered on BHK cells as the best available assay, as these cells form a monolayer; in contrast, PC12 cells, and most neuronal cell lines, do not form a monolayer. Thus, the titers obtained on BHK cells are higher than the titers obtained on PC12 cells [5,53]. Expression from the TH or INS-TH-NFH promoter in fibroblast cells is ectopic expression, and this inappropriate expression declined rapidly at longer times after gene transfer (not shown). wt HSV-1 was not detected in these vector stocks (<10 plaque forming units (pfu)/ml).

We previously used PCR assays to quantify the vector genomes (VG)/ml of vectors packaged using either gC – GNDF or gC – BNDF [21]. The VG/ml of these stocks were similar to those obtained using wt gC; the ratio of VG/IVP ranged from 1 to 45, and wt gC supports a ratio of ~10. We did not repeat the assay for VG/ml, because the present study applied the same packaging conditions to different vectors.

### Gene transfer experiments in the brain

These studies were approved by the West Roxbury VA Hospital IACUC. Male Sprague Dawley rats (175–200 gm) were used for these experiments. Appropriate volumes of pTHlac/gC – bdnf and pHSVpkcΔGG/gC – bdnf were mixed together to yield a titer of 1.8 × 10^6 ^IVP/ml for each vector, and appropriate volumes of pINS-TH-NFHlac/gC – gdnf and pHSVpkcΔGG/gC – gdnf were mixed together to yield a titer of 5.0 × 10^5 ^IVP/ml for each vector. Each mixture of vector stocks was delivered by stereotactic injection (2 sites, 3 μl/site) into the midbrain proximal to the substantia nigra of the right hemisphere (anterior-posterior (AP) -5.5, medial-lateral (ML) -1.9, dorsal-ventral (DV) -7.1; AP -5.5, ML -2.3, DV -6.8) [21]. AP is relative to bregma, ML is relative to the sagittal suture, and DV is relative to the bregma-lambda plane [54]. Injections used a micropump (model 100, KD Scientific); the 3 μl innoculum was injected over ~5 minutes, the needle was maintained in place for an additional 5 minutes, and then slowly withdrawn over ~5 minutes. Rats were perfused, brains were sectioned, and immunofluorescent costaining was performed, as described [5]. Alternating sections were costained with either mouse anti-β-gal (1:200 dilution) or mouse anti-flag (detects PkcΔGG, 1:1,000 dilution) and rabbit anti-TH (1:1,000 dilution). Primary antibodies were visualized using rhodamine isothiocyanate-conjugated goat anti-mouse IgG and fluorescein isothiocyanate-conjugated goat anti-rabbit IgG.

### Cell counts

Twenty-five μm coronal sections that contained the midbrain were prepared, and recombinant gene products were detected in ~20 of these sections. Alternating sections were analyzed for costaining of either β-gal-IR or PkcΔGG (flag-IR) and TH-IR. Photomicrographs were taken under 60× magnification using a video camera. The positive cells (β-gal-IR or flag-IR) in each section were scored for costaining with TH-IR, and all the positive cells in each section were scored. Each section was counted at least two times, on different days, and the two values differed by <10% for each section.

### Statistical analyses

Statistical comparisons were performed using ANOVAs (Sigmastat).

## Abbreviations

AP, anterior-posterior; β-gal, β-galactosidase; BDNF, brain-derived neurotrophic factor; DL, dorsal-ventral; EPO, erythropoietin; GAD, glutamic acid decarboxylase; GDNF, glial cell line-derived neurotrophic factor; GFAP, glial fibrillary acidic protein; HSV-1, Herpes Simplex Virus; HVEM, herpesvirus entry mediator; IE, immediate early; Ig, immunoglobulin; INS, insulator; IR, immunoreactivity; ML, medial-lateral; NF-H, neurofilament heavy gene; pfu, plaque forming units; PKC, protein kinase C; SNc, substantia nigra pars compacta; TH, tyrosine hydroxylase; VG, vector genomes; X-Gal, 5-bromo-4-chloro-3-indoyl-β-D-galactopyranoside.

## Authors' contributions

HC performed the vast majority of this study; she was assisted in the vector packaging by XW and in the histology by LK. GZ performed the gene transfer, perfusions, and sectioning. HC and AIG conceived of the study, and participated in its design and coordination. AIG wrote the manuscript. All authors read and approved the final manuscript.
